# Supplementary material for: Sex difference in pathology of the ageing gut mediates the greater response of female lifespan to dietary restriction
Source: eLife. 2016 Feb 16;5:e10956. doi: 10.7554/eLife.10956 (PMC4805549; doi:10.7554/eLife.10956)
Supplement: Figure 3—source data 1. — DOI: http://dx.doi.org/10.7554/eLife.10956.009 [file elife-10956-fig3-data1.docx]

**Figure 3 - Source Data**

**Cox Proportional Hazards Lifespan Analysis**

|  | *_log_*_(hazard ratio)_ | _hazard ratio_ | _S.E.M_  _(log(hazard ratio))_ | *_z_* | *_p_* | _significance_ |
| --- | --- | --- | --- | --- | --- | --- |
| _food_ | _0.84402_ | _2.3257_ | _0.09308_ | _9.068_ | _< 2e-16_ | _***_ |
| *_NP1>tra_* _vs_ *_NP1>w, tra>w_* | _-0.01427_ | _0.98583_ | _0.10537_ | _-0.135_ | _0.8923_ |  |
| *_NP1>w_* _vs_ *_tra>w_* | _0.13883_ | _1.14892_ | _0.1638_ | _0.848_ | _0.3967_ |  |
| _gender (male)_ | _2.05524_ | _7.80873_ | _0.19841_ | _10.359_ | _< 2e-16_ | _***_ |
| _response to food;_ *_NP1>tra_* _vs_ *_NP1>w, tra>w_* | _-0.04061_ | _0.9602_ | _0.06718_ | _-0.604_ | _0.5455_ |  |
| _response to food;_ *_NP1>w_* _vs_ *_tra>w_* | _-0.00055_ | _0.99945_ | _0.10704_ | _-0.005_ | _0.9959_ |  |
| _response to food; sex_ | _-0.49333_ | _0.61059_ | _0.12344_ | _-3.997_ | _6.43E-05_ | _***_ |
| *_NP1>tra_* _vs_ *_NP1>w_*_,_ *_tra>w_* _(males)_ | _-0.10932_ | _0.89645_ | _0.14241_ | _-0.768_ | _0.4427_ |  |
| *_NP1>w_* _vs_ *_tra>w_* _(males)_ | _-0.09663_ | _0.90789_ | _0.22414_ | _-0.431_ | _0.6664_ |  |
| _response to food;_ *_NP1>tra_* _vs_ *_NP1>w, tra>w_* _(males)_ | _0.19541_ | _1.21581_ | _0.08991_ | _2.173_ | _0.0298_ | _*_ |
| _response to food;_ *_NP1>w_* _vs_ *_tra>w_* _(males)_ | _0.07425_ | _1.07708_ | _0.14391_ | _0.516_ | _0.6059_ |  |
